# Progression of Vision in Chinese School-Aged Children Before and After COVID-19

**DOI:** 10.3389/ijph.2022.1605028

**Published:** 2022-08-11

**Authors:** Wenjing Wang, Shuzhen Peng, Faxue Zhang, Boya Zhu, Longjiang Zhang, Xiaodong Tan

**Affiliations:** ^1^ School of Public Health, Wuhan University, Wuhan, China; ^2^ People’s Hospital of Huangpi, Wuhan, China

**Keywords:** children, COVID-19, prevalence, spherical equivalent refraction, myopia progression

## Abstract

**Objectives:** To investigate the changes of vision, including the prevalence of myopia, hyperopia, poor vision, and the spherical equivalent refraction (SER), in school-aged children before and after the pandemic of Coronavirus Disease 2019 (COVID-19).

**Methods:** A school-based vision screening study was performed on children in 133 primary schools in Wuhan. This study was conducted in 4 consecutive years (2018–2021).

**Results:** A total of 468,094 children (936,188 eyes) were recruited, 255,863 (54.7%) were boys. The SER decreased in 2020 compared to other years after the age of 10. A positive myopia shift was found in younger children aged 6 (0.1 D), 7 (0.05D), and 8 (0.03 D) in 2020 compared to 2019. The progression of vision has improved slightly in 2021. Among the students included in the study, 33.7% were myopia.

**Conclusion:** The vision of older children decreased significantly during the COVID-19. After the pandemic, there is still a high risk for them. In the future, the focus on vision prevention and control should move forward to preschool children.

## Highlights

• This school-based study examined the changes of vision, including the prevalence of myopia, hyperopia, poor vision, and the spherical equivalent refraction (SER), in children (468,094) aged 6–15 years before and after the COVID-19 pandemic.
• In this study, we found that the vision of older children decreased significantly during the COVID-19. After the pandemic, there is still a high risk for them.
• For younger children, they will have more opportunities to control and improve vision. In the future, the focus on vision prevention and control should move forward to preschool children.

## Introduction

Good vision development is essential for teenagers. As we all know, myopia is a major global public health issue around the world, which is the leading cause of severe vision impairment. It is estimated that 1.4 billion people were myopic in 2000, and it is predicted to reach 4.8 billion by 2050 [1]. One study shows that the prevalence of myopia is increasing year by year [2]. Besides myopia, hyperopia, and poor vision should also be concerned.

Evidence suggests that school-age students are a particularly vulnerable group, as refractive errors and myopia are prevalent among school-age students [3]. A study from a hospital in Nepal (South Asia) reported that the prevalence of myopia in children was 47.16% [4]. The results of other literature suggested that the myopia was prevalent in 36.4% children of 8 years old in Taiwan, China in 2016, while the prevalence of myopia was only 2.4% in the Netherlands among 6 years old children in 2018 [5, 6]. The prevalence of poor vision among school-age students is also high [7]. And poor vision has detrimental effects on students’ academic performance, motor skills, physical and mental health [8].

In December 2019, the COVID-19 rapidly spread in China and around the world. In response to the epidemic, the Chinese government began closing schools nationwide school closure at the end of January 2020 as an emergency measure to prevent the spread of infection [9]. According to the UNESCO, as of early September, 820 million children and youth had been affected by school closures. And schools in forty-six countries have closed nationwide [10]. Several studies have demonstrated that being forced to study at home due to school closures may have a negative impact on children’s physical and mental health [11]. These effects may be detrimental to the development of children’s vision. One study showed that children were at higher risk of myopia progression during COVID-19, which was related to long-term online learning and digital screen reading [12]. The results of a population-based study showed that the outdoor time of school-age children in Hong Kong significantly reduced, the screening time increased, and the incidence of myopia may potentially increase. And childhood myopia, as a result of COVID-19, is a potential public health crisis [13].

Wuhan, once the hardest-hit area of COVID-19, may have a greater impact on primary school children’s vision. Therefore, the present study used longitudinal data from the Wuhan Center for Adolescent Poor Vision Prevention and Control to investigate the changes of vision in school-aged children in China during the COVID-19. SER change, myopia and hyperopia incidence, and poor vision were calculated and compared between September 2018 to June 2021.

## Methods

### Study Design

The study was completed under the management of the Education Bureau, based on the vision health management work in Wuhan. Wuhan’s vision health management was implemented in 2007 and became the “National Youth vision health management demonstration area” in 2018. Wuhan Center for Adolescent Poor Vision Prevention and Control is devoted to regularly monitor students’ vision every year. The Center will also carry out comprehensive vision interventions for students, including interventions on healthy behaviors, visual environment, and eye physiology. Furthermore, the center cooperates with schools to propagate knowledge of vision health and advocate eye protection. And it is committed to making schools, families, and students pay attention to and solve the vision problem of teenagers.

A stratified cluster approach is adopted to complete the study. In total, data were collected from 133 schools in 14 districts of Wuhan. The information about age and gender of each student is included in the dataset, as well as their eyes’ ocular parameters, like uncorrected visual acuity (UCVA), poor vision, spherical equivalent refraction (SER), and hyperopia.

This school-based study was approved by the ethics board of the School of Medicine, Wuhan University, China.

The study started only after parents of all the participants had given their written consent.

### Procedures

From September 2018 to June 2021. With the cooperation of school teachers, the Wuhan Center for Adolescent Poor Vision Prevention and Control organized professional personnel to investigate primary school students in Wuhan.

The uncorrected visual acuity information is carried out using a standard logarithmic visual acuity E chart under the supervision of an ophthalmologist. The testing distance between our subjects and the test chart is 5 m. Line 0 log MAR is the start of the test. Once the correctly identified of the visual acuity chart has been done by subjects, the ophthalmologist will point to the next smaller line. On the contrary, the bigger line will be pointed to by the doctor. All students should respond within 3 s and are supposed to keep their eyes relaxed. After that, 5% of the participants are randomly selected for re-examined their uncorrected vision. Auto-refractor (TOPCON ACP-8) is used to measure the Non-cycloplegic refraction. And the ametropia is tested in each naked eye by the DK-10 phoropter.

Exclusion criteria are as follows: wearing contact lenses or; suffering from eye disease; and having a history of ocular surgery. Before the test, the children will be questioned about the exclusion criteria. And the data were excluded from the analysis If any of the above situations are encountered.

### Definitions

The spherical equivalent refraction (SER) is calculated as the dioptric powers of the sphere and half of the cylinder (sphere +0.5 × cylinder). Myopia was defined as SER of less than −0.5D [14]. And subjects wearing orthokeratology lenses are identified as myopic. Hyperopia was defined as greater than +0.5D. And poor vision is defined as the visual acuity below 5.0 by using the standard logarithmic visual acuity chart.

### Statistical Analysis

Annual vision screening data are presented as mean values of SER. The analysis was performed using R and Rstudio software. The figures were prepared using Origin 2021. Chi-square test was used in different groups when appropriate. *p* < 0.05 (two-sides) was considered as significant at statistics.

## Results

### Study Participants

A total of 468,094 test results (936,188 eyes) were analyzed in this study, 255,863 (54.7%) subjects were boys. The age range was 6–15 years old with a mean age of 8.9 ± 1.68 years. Among the students included in the study, 33.7% were myopic, 11.0% were hyperopia, and 45.7% were poor vision. The mean uncorrected visual acuity of the right and left eyes was 4.92 ± 0.26, and 4.93 ± 0.25 respectively.

### The Spherical Equivalent Refraction

We reported the mean SER of the 4 screening years in Table 1. We found that the mean SER findings were relatively stable in school-aged children aged 6–9 from 2018 to 2021. And students in this age group were not affected by the epidemic in 2020. The results showed that there had a substantially decreased of SER in 2020 compared to other years after the age of 10, especially for children aged 12 (−1.75 D) and 13–15 (−1.84 D) years. What’s worse, the vision continued to decline in 2021 among children aged 12–15. In addition, there were 601 children with at least 1 eye refraction out of range (<−8.00D); of these, 346 children had both eye refractions out of range.

**TABLE 1:** Spherical equivalent refraction values during each year in students (Wuhan, China, 2021).

Age, Y	No.	SER, mean (SEM)			
		2018	2019	2020	2021
6	23,585	−0.13 (0.80)	−0.19 (0.76)	−0.09 (0.77)	0.01 (0.66)
7	97,506	−0.18 (0.84)	−0.23 (0.82)	−0.18 (0.85)	−0.15 (0.91)
8	91,742	−0.33 (0.94)	−0.45 (0.96)	−0.42 (0.98)	−0.38 (1.07)
9	85,730	−0.53 (1.08)	−0.70 (1.12)	−0.68 (1.14)	−0.69 (1.23)
10	71,342	−0.80 (1.27)	−1.01 (1.31)	−1.04 (1.37)	−1.03 (1.44)
11	63,226	−1.16 (1.57)	−1.33 (1.50)	−1.41 (1.59)	−1.41 (1.62)
12	30,991	−1.46 (1.74)	−1.53 (1.63)	−1.75 (1.70)	−1.76 (1.82)
13–15	3,971	−1.10 (1.49)	−1.56 (1.60)	−1.84 (1.92)	−1.93 (1.87)

SER, spherical equivalent refraction.

**FIGURE 1:**
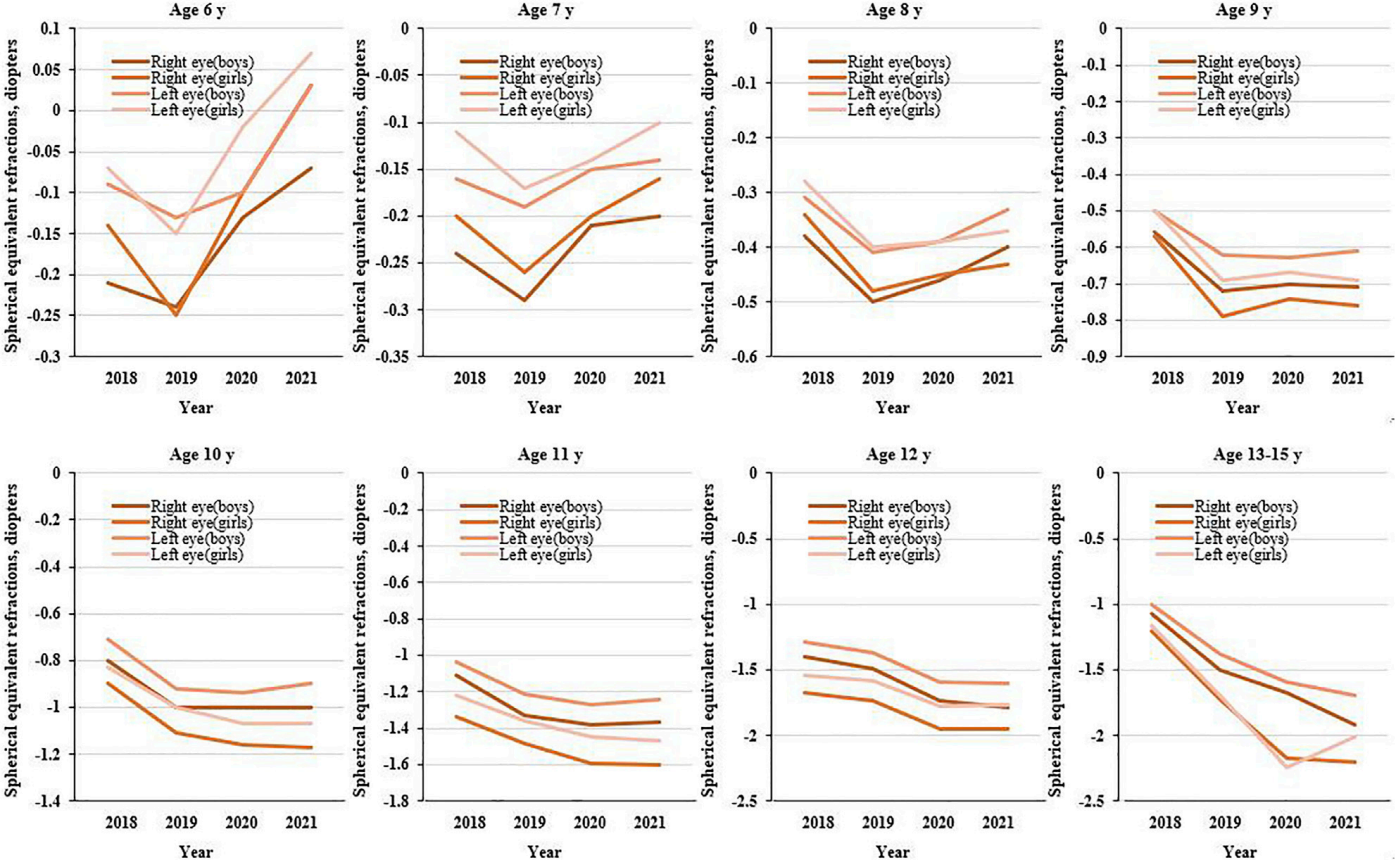
Mean spherical equivalent refraction for primary school students aged 6–13 years during the 4 years of screenings (Wuhan, China, 2021).

After analyzing both eyes and gender among students of the data, we found that girls were more likely to suffer from myopia than boys, and the right eye was more myopic than the left (Figure 1).

### The Prevalence of Myopia and Hyperopia

**FIGURE 2:**
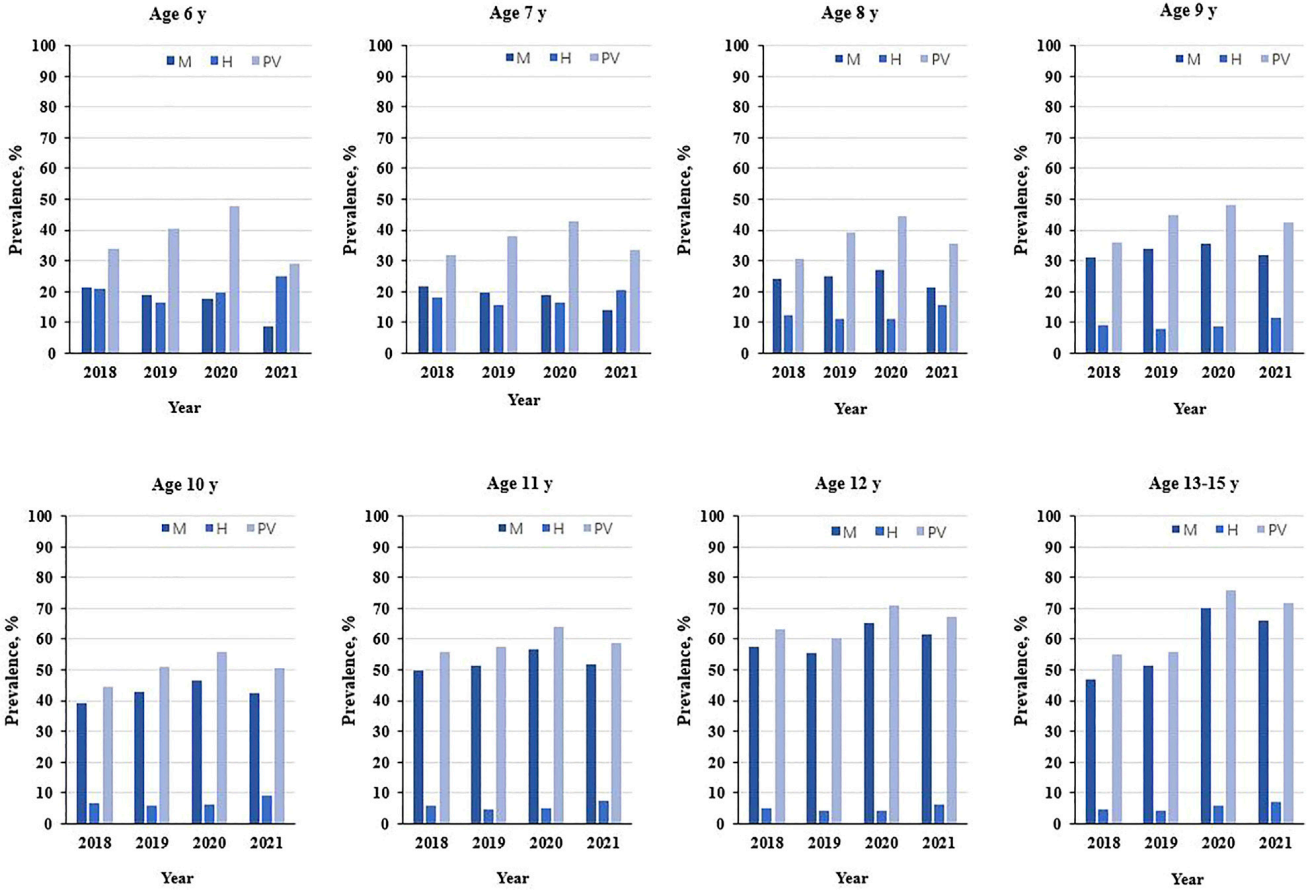
Progression of vision of primary school students aged 6–15 years during the 4 years of screenings (Wuhan, China, 2021).

The prevalence of myopia and hyperopia from 2018 to 2021 is reported in Table 2 and Figure 2. In the present study, we can observe that the older age groups (9–13 years) had a substantial increase in the prevalence of myopia. The prevalence of myopia in 2020 was 65.2% at 12 years and 70.3% at 13–15 years. These results were significantly higher than the rate of myopia in 2019: 55.3% at 12 years and 51.3% at 13–15 years. From 2018 to 2021, the rate of myopia among the first graders decreased year by year, while the rate of third to sixth-grade students gradually increased, with the highest myopia rate in 2021.

**TABLE 2:** Progression of vision for each year in school-aged children (Wuhan, China, 2021).

Grade	No.	Prevalence per year, %												*p* Value		
		2018			2019			2020			2021					
		M	H	PV	M	H	PV	M	H	PV	M	H	PV	M	H	PV
1	99,736	21.1^a^	20.1	32.5	18.3	18.9	40.2	16.8	18.4	43.8^a^	13.8	20.5^a^	33.4	<0.001	<0.001	<0.001
2	94,806	22.6	13.6	29.9	20.0	12.9	35.1	24.4^a^	12.4	43.8^a^	22.5	14.9^a^	36.0	<0.001	<0.001	<0.001
3	87,852	28.8	9.5	34.2	27.9	8.7	40.7	32.4	9.4	46.2^a^	33.9^a^	10.5^a^	44.1	<0.001	<0.001	<0.001
4	71,315	38.3	6.6	42.4	37.9	6.1	47.0	43.8	6.8	54.1^a^	44.2^a^	8.8^a^	52.2	<0.001	<0.001	<0.001
5	66,018	46.1	6.0	52.9	47.8	4.9	54.5	53.6	5.5	61.1^a^	53.7^a^	6.9^a^	60.3	<0.001	<0.001	<0.001
6	48,316	58.2	4.6	63.8	56.9	3.9	62.0	64.0	4.2	69.7	66.0^a^	5.8^a^	71.0a	<0.001	<0.001	<0.001

a Highest prevalence within the age group during the 2018–2021 screening.

M, myopia; H, hyperopia; PV, poor vision.

The hyperopia rate of primary school students was relatively stable, reaching the highest every year in 2021. Moreover, the prevalence of hyperopia decreased with the increase in grade. From grade 1 to grade 6, the prevalence of myopia and hyperopia were statistically significant between the different years.

### The Prevalence Rate of Poor Vision

The prevalence rate of poor vision tended to increase significantly with the increase of age. Among different age groups, the rate was the highest in 2020 and decreased in 2021. In addition, we found that the prevalence of poor vision findings was relatively stable among children aged 9 to 15 from 2018 to 2021 (Figure 2).

Vision screening results showed that elementary school pupils had the highest rate of poor vision loss in 2020 compared to other years, especially among children in grades 5 and 6. The prevalence of poor vision tended to increase significantly with the increase in grade. However, the first graders were a special case, with a higher rate of poor vision than the second graders. In 2021, the prevalence of poor vision among children in grades 1 and 2 decreased by about 10 percent. From grade 1 to grade 6, the prevalence of myopia and hyperopia were statistically significant between the different years, shown in Table 2.

## Discussion

This vision screening project was launched in Wuhan in 2007 to monitor and prevent myopia in young people. And it was refined in 2018. The COVID-19 pandemic in 2020 has caused the vision decline of students in many cities, and the myopia rate has significantly increased [15]. Nevertheless, elementary school students in Wuhan, the epicenter of the outbreak, may be more affected. Wuhan Center for Adolescent Poor Vision Prevention and Control continuously monitored and controlled the myopia of children. As a result, their vision will be different in 2021 from that of the past.

The study shows that the SER distribution among students has a different trend around the age of 10 from 2015 to 2019. In 2020, a substantial myopic shift (approximately -0.25D) was found in students aged 12–15 years, and approximately 0.08D for children aged 6–7 years. The myopic shift increased with age at 10–15 years. However, among students aged 6–9, myopic shift showed the opposite trend. School-age children confined to home from January to May due to COVID-19 in 2020, and online courses were offered. Evidence from the literature showed that the pandemic has led to less outdoor activity time and more online classes for teenagers. Short-term outdoor activities and near work are risk factors for myopia. And the rate of myopia among the students has increased significantly [16, 17]. While for children aged 6–9, their learning tasks are not heavy, online courses are not long [18]. Simultaneously, the Center united schools to promote the knowledge of vision health and raise parents’ awareness of eye care. Therefore, parents will control screen time and increase the time for outdoor activities to protect their children’s eyes. A recent randomized controlled trial showed that outdoor activity can inhibit disease progression by 30% within 1 year in myopic children aged 6–7 years. We also observed that boys had better vision than girls, and the vision in the left eye was better than the right eye. This finding was consistent with prior studies [19]. The current research has not found that there is a genetic relationship between myopia and gender, and the gender difference in myopia may be related to eye development and behavioral habits [20]. In the other studies, the visual difference between right and left eyes may be related to ocular structural variations or ocular dominance [21, 22]. A study has reported that the nondominant eye may have a lower degree of myopia than the dominant eye among individuals with anisometric myopia [23]. In previous reports, approximately one-third of the population was left ocular dominant and two-thirds were right ocular dominant [24]. However, the specific reasons still require further investigation, especially the research on primary school students.

The myopic shift of SER may coincide with the change in the prevalence of myopia in student aged 6–7 declined in 2020, while the myopia rate of pupils in higher grades increased year by year. Maybe higher graders were more stressed, spending more time reading and writing at home, and are more likely to take extra classes. So, they have less outdoor time and more digital screen time [13]. Besides, sleep duration can also affect the progression of myopia. Someone found that short sleep is an independent risk factor for myopia. Children who slept 7 h or less, or around 8 h were at a higher risk compared to children who slept 9 h or more daily [25]. Admittedly, students in grades 5 and 6 spend more time studying than sleeping, so they have a higher rate of myopia. Myopia usually occurs between the ages of 6–8, with a faster rate of progression and axial length elongation in this age group [26]. The plasticity of myopia is high and myopia control may be easier within this age window. While the plasticity of myopia is low and myopia control is harder beyond this age window [27]. Therefore, monitoring and managing vision in young children is of great significance.

In this study, the one-year prevalence of myopia was around 15% among grade 1 students, which is lower than the research in the southwest part of China (33.6%) [28]. However, considering that a small number of students are short-sighted when entering school, the focus on vision prevention and control should be moved forward to preschool children. A 3-year cohort report suggested that there were 142 myopic (63.7%) students from Beijing Myopia Progression Study. Their myopia rate was higher than that of grades 5–6 in our study [29]. Nevertheless, some countries have low prevalence rates, such as Brazil of3.14% and Saudi Arabia of 0.7% [30, 31]. The prevalence of hyperopia in Wuhan was higher than in other areas, such as India, Iran, and Shanghai, while lower than in Qinghai [32–35]. In addition, because the prevalence of hyperopia is relatively low and children are sensitive to environmental factors, they are prone to myopia and experience rapid myopia progression. As a result, the older primary students had a lower hyperopia rate [35].

Consistent with a previous study, the prevalence of poor vision increased dramatically with grades. This showed that the trend of poor vision was related to the increasing age of young people [36]. In this study, the prevalence rate of poor vision among children was higher in 2020 than in other years. Epidemiological results have indicated a high prevalence of low vision among school-age students due to long-term visually demanding academic tasks and long-term indoor sedentary lifestyles [37]. In addition to the learning burden, age, sleeping or TV time, and the time spent on homework were also positively associated with poor vision in students aged 9–18 [38]. Therefore, the eyesight of children had declined after COVID-19 home confinement. And the prevalence of poor vision is higher than in a study of 170,000 students [7]. It is noteworthy that the rate of poor vision of students has decreased by 2021. And the younger the age, the more the percentage of decline. This may benefit from the vision health management in Wuhan: public policies to control myopia are closely integrated with the education system. They strengthened parents’ education on the incidence and development of myopia, instructed students on good eye habits during the pandemic lockdown and beyond, and incorporated outdoor activities into school time, and more [39]. These measures are similar to some countries in East Asia, such as Taiwan and Singapore [40].

This was a large-scale school-based study providing the current status of myopia prevalence in different age groups among primary school children. Furthermore, this was a long-term survey, which showed the progression of vision for 4 years before and after COVID-19. Even so, it has some limitations. First, our study did not use cycloplegia, and it is known that cycloplegic refraction yields is better than non-cycloplegic autorefraction. However, due to a large number of students and limited resources, cycloplegic refraction was difficult to apply to each student. Second, the relatively small sample size of grade 6 may introduce bias to the vision results. Third, this study did not have data on ocular biometric parameters, such as lens thickness, anterior chamber depth, or corneal curvature. Fourth, the population tested varies from year to year due to graduating and newly admitted students each year, and so these differences reflect population averages. Fifth, some other factors that affect the state of vision of students may not be considered.

### Conclusion

We showed the acceleration of development and progression of vision among older (10–13 years) schoolchildren in Wuhan during the COVID-19. While for younger individuals (6–8 years), they are in an important period for the progression of myopia. And they may have more chance of having their vision corrected than older children. Moreover, preschool children should also be one of the key groups for vision prevention and control. After the pandemic, there was a slight improvement in vision. It is suggested that the COVID-19 pandemic may remain a threat to the development of myopia in students. Further studies are needed to assess the generalizability of these findings and the long-term follow-up of these children.

## Data Availability

The data that support the findings of this study are available from the corresponding author upon reasonable request.
